# Clinical and pathological characteristics of blastoid mantle cell lymphoma: a single institution experience

**DOI:** 10.12688/f1000research.149582.2

**Published:** 2024-07-26

**Authors:** Vidya Monappa, Swathi Prabhu, Ranjini Kudva, Vishwapriya Mahadev Godkhindi, Kanthilatha Pai, Ananth Pai, Sharada Mailankody

**Affiliations:** 1 Department of Pathology, Kasturba Medical College, Manipal, Manipal Academy of Higher Education, Manipal, Karnataka, 576104, India; 2 Department of Medical Oncology, Kasturba Medical College, Manipal, Manipal Academy of Higher Education, Manipal, Karnataka, 576104, India

**Keywords:** Mantle cell lymphoma, blastoid, cyclin D1, lymphoma

## Abstract

**Background:**

Blastoid mantle cell lymphoma (B-MCL) is a rare aggressive lymphoma. It is characterized by blastoid morphology with high proliferation and inconsistent immunohistochemistry (IHC), making it a diagnostic challenge for the pathologist.

**Methods:**

This is a retrospective analytical cohort study. We reviewed biopsy confirmed cases of B-MCL diagnosed over a period of 10 years (January 2012 to December 2022). The clinical presentation, histopathological and IHC findings, treatment received, and survival outcomes were studied. Randomly selected cases of classic MCL (n=12), diagnosed during the same period served as controls.

**Results:**

A total of 12 cases were studied. Four cases were transformed from previously diagnosed MCL; 8 cases arose *de novo.* Mean age was 61.17 years and the male: female ratio was 5:1. Half of the cases showed extra nodal extension and 81.8% had bone marrow involvement. Gastrointestinal tract was the most common site of extra nodal involvement. Histopathological examination showed diffuse involvement of the lymph node with medium sized cells. On immunohistochemistry, one of the cases showed loss of CD5 expression while the other had aberrant CD10 expression. Mean Ki-67 index was 58.09% in the cases and 16.33% in controls and was statistically significant ( *p*=0.005). The median overall survival (OS) for cases was 2 years vs 8 years in controls. The p53 over expression (>30% nuclear positivity) was seen in 66.6% cases (4/6).

**Conclusion:**

There are several factors that contribute to the aggressiveness of B-MCL, and new treatment approaches might be required to improve patient outcomes.

## Introduction

Mantle cell lymphoma (MCL) is a known subtype of B-cell non-Hodgkin lymphoma (NHL), with an aggressive clinical course and outcome. Its incidence is higher in Asians than in western population. ^1^^,^^2^ It was first described in the year 1973 when Karl Lennert et al. used the term centrocytic lymphoma due to its resemblance to centrocytes. In 1992, Banks et al. coined the term MCL, which was entrenched in the WHO blue book in 2001, officially recognizing this entity. ^3^ It was named so because of its distinctive growth pattern, called “mantle zone pattern,” wherein, tumor cells encircle and overcrowd the reactive germinal center, replacing it. ^4^ It commonly affects elderly male patients in their 7 ^th^ decade. Nodal involvement is the most common presentation. However, extra nodal involvement, especially of the gastrointestinal tract, bone marrow, and spleen, is not unusual. MCL molecular pathogenesis is characterized by the signature translocation t (11;14) (q13; q32), involving immunoglobulin heavy chain (IGH) and *CCND1* genes (encoding cyclin D1 and regulating the CDK4/6 complex with pro-tumorigenic effects). Apart from this characteristic translocation, SOX11 overexpression, *p53* mutations, and lymph node microenvironment contribute to the proliferation and growth of mantle cells attributing to its malignant behavior and aggressive nature. ^5^

MCL accounts for 3 to 10% of NHL of which 1/3 ^rd^ is blastoid subtype (B-MCL), making it one of the rarest lymphoma subtypes. ^6^^,^^7^ B-MCL could arise *de novo* or transform from a classic subtype, although the former is more common. It is classified by its distinct morphological and immunohistochemical features, which contribute to its aggressive behavior, associated with significant mortality and morbidity. ^2^ The pattern of lymph node involvement in B-MCL varies, predominantly diffuse or predominantly nodular, with a few cases exhibiting a mantle zone-like pattern. Neoplastic cells resemble lymphoblasts with high mitotic activity (≥20–30/10 high-power fields). ^6^ There is no consensus on the treatment of B-MCL. They are usually detected as stage III/IV disease and are managed aggressively owing to the high incidence of relapse rates. The treatment regimen is similar to MCL, with a few modifications depending on the patient status, stage at presentation, and affordability factors.

In this study, we reviewed 12 cases of biopsy-proven B-MCL and discussed their clinical presentation, morphological and immunohistochemical findings, treatment received, and survival outcomes. Our aim was to identify factors (clinical and pathological) that differentiate B-MCL from classic MCL. This experience will help clinicians devise better treatment strategies, resulting in improved patient survival rates.

## Methods

This was a retrospective analytical cohort study. All biopsy-confirmed cases of B-MCL at our institution over a 10-year period (January 2012 to December 2022) were included. The clinical presentation, histopathological and immunohistochemical findings, treatment approaches, and survival outcomes of the patients were studied. Data were extracted from archival and electronic medical records (EMR). A total of 12 B-MCL cases were included in this study. Randomly selected cases of classic MCL (n=12) diagnosed within the same time frame were used as controls. Clinicopathological parameters analyzed included age, sex, nodal and extra nodal involvement, bone marrow involvement (stage IV), and hepatosplenomegaly. B symptoms were included if they were documented previously. The date of diagnosis, treatment received, alive, dead, or lost to follow-up, and any event (relapse/progression) were recorded to facilitate survival studies. Patients with insufficient details were excluded from this study.

### Immunohistochemistry (IHC)

Lymphoma panels for CD3, CD5, CD20, CD10, BCL6, BCL2, Ki-67, cyclin D1 were performed in the majority of cases and CD23, TdT, and p53 in selected cases. Loss of IHC markers or aberrant expression, if any, was documented to identify deviations from the expected expression pattern. Ki-67 was assessed by counting the percentage of nuclear positivity in 10 consecutive high-power fields (HPF) in hotspot areas. CD10 and BCL6 were reported to be positive if ≥30% of the cells showed nuclear positivity. BCL2 with ≥50% nuclear expression in the tumor cells was taken as positive. The p53 IHC with nuclear expression >30% was considered as p53 over expression and <30% as low expression, as per the guidelines of the Nordic lymphoma study group. ^8^

### Statistics

The collected data were analysed using IBM SPSS Statistics for Windows, Version 29.0 (Armonk, NY: IBM Corp). Descriptive statistics, frequency analysis, and percentage analysis were used for categorical variables, and the mean and standard deviation (S.D.) were used for continuous variables. To find a significant difference between the bivariate samples in the independent groups, an independent sample t-test was used. To predict the survival rate, the Kaplan-Meier curve was used with the log-rank method. To find the significance in qualitative categorical data, the chi-square test was used similarly; if the expected cell frequency was less than 5 in 2×2 tables, Fisher’s exact test was used. In all the above statistical tools, a probability value of 0.05 is considered as significant level.

## Results

There were 12 cases of biopsy and IHC confirmed B-MCL included in this study. Their ages ranged from 43 to 80 years, with a mean Age at diagnosis of 61.17 years. There were 10 males and 2 females with a male: female ratio of 5:1. Among the 12 cases, four cases (33.3%) were found to have progressed from previous classic MCL, while the remaining 8 cases arose *de novo.* There were no statistical differences in age or sex when compared with the controls.

All the patients exhibited nodal involvement. B-MCL cases showed higher rates of extra nodal involvement (50%, n=6/12) when compared to classic MCL (8.3%, n=1/12). Extra nodal sites included the gastrointestinal tract (n=3, 50%), nasopharynx (n=2, 33.3%), and central nervous system (CNS) (n=1, 16.6%). Bone marrow involvement was noted in 9/11 cases and 9/12 controls, respectively. B symptoms were observed in 6/10 cases (60%) while hepatomegaly (n=6/12, 50%), splenomegaly (n=5/10, 50%) were observed more frequently in cases than controls. However, these differences were not statistically significant. The demographic and clinical characteristics of the patients and controls are shown in Table 1.

**Table 1.** Demographic and clinical characteristics of cases and controls.

Parameters	Cases (N=12)	Controls (N=12)	P value
Age (yrs)	Mean, 61.17	Mean, 62.50	0.766
Sex	M:5 F:1	M:11 F:1	1.000
Nodal involvement	12 (100%)	12 (100%)	1
Extra-nodal involvement	6 (50%)	1 (8.3%)	0.024
Stage IV at diagnosis (Bone marrow involvement)	9/11 (81.8%)	9/12 (75%)	1.000
B-Symptoms	6/10 (60%)	3/8 (37.5%)	0.637
Hepatomegaly	6 (50%)	3/7 (30%)	0.415
Splenomegaly	7 (58.3%)	5/10 (50%)	0.696

### Histomorphology

Effacement of nodal architecture was observed in all cases. Predominantly diffuse pattern (<50% nodular) was observed in 91.6 % of B-MCL(n=11/12), with one case showing predominantly nodular growth pattern (n=1/12,8.4%). Similar growth pattern was observed in control cases as well with slightly lower percentages of predominantly diffuse pattern (n=8/12, 66.6%) and nodular (n=4/12, 33.3%). The *p* value was not statistically significant ( *p*=0.131), and none of our cases had a mantle zone pattern.

Small lymphoid cells with slightly irregular nuclear contours and scant cytoplasm were observed in all the classic MCL cases. B-MCL cases showed medium-sized cells with oval nuclei, stippled chromatin, inconspicuous nucleoli, and a scant cytoplasm. This morphology is similar to that of diffuse large B-cell lymphoma (DLBCL) or lymphoblastic lymphoma. Scattered hyalinized blood vessels and histiocytes were observed in both cases and controls. B-MCL demonstrated a high mitotic rate in all cases, exhibiting brisk mitosis (>20/10HPF). In contrast, the control group showed only occasional mitotic figures, and this difference was statistically significant ( *p*<0.0001). Focal necrosis was seen in 2/12 cases (16.6%) and 1/12 controls (8.3%) and was not statistically significant ( *p*=0.537). The histomorphological features of B-MCLs and classic MCL are shown in Table 2.

**Table 2.** Histopathological characteristics of B-MCL vs classic MCL.

Histopathological features	Cases (n=12)	Controls	P value
Architecture			0.131
• Diffuse (>50%)	11 (91.6%)	8 (66.6%)	
• Nodular (>50%)	1 (8.3%)	4 (33.3%)	
Cell size			
• Small	0	12 (100%)	<0.00001
• Medium	12 (100%)	0	
Brisk mitosis	12 (100%)	0 (0%)	<0.00001
Necrosis	2 (16.6%)	1 (8.3%)	0.537
Pleomorphic cells	1 (8.3%)	0 (0%)	NA

### Immunophenotyping

All cases were CD20 positive and CD3 negative. CD5 expression was lost in one case (n=1, 8.3%) and aberrant CD10 expression was noted in another case (n=1, 8.3%). All cases and controls showed overexpression of cyclin D1. Furthermore, BCL2 was positive in 83.3% (n=5/6) cases and 100% of controls (n=6/6). BCL6 expression was negative in all cases and controls. CD23 and TdT were performed in a few patients (3 cases, 1 control;4 cases, 2 controls respectively) and were negative.

The Ki-67 proliferation index was significantly higher (range, 40 to 90%; mean of 58.1%) in the cases compared to the controls (range, 2% to 33%; mean of 16.3%) ( Figure 2). This finding was statistically significant, with *p* value of 0.005. This corresponded to the brisk mitosis noted on histopathology of B-MCL. p53 IHC was available for six cases. Over expression (>30%) nuclear positivity was seen in 4/6 cases (66.6%), while the other two cases showed <30% nuclear positivity ( Figure 3). Table 3 highlights the IHC findings in our cases (B-MCL) and the controls (classic MCL).

**Table 3.** Immunohistochemical characteristics of B-MCL vs classic MCL.

IHC markers		Cases (N=12)	Controls (N=12)	p value
CD5	positive	11 (91.6%)	12/12 (100%)	0.450
	negative	1 (8.3%)	0/10 (0%)	
CD10	positive	1 (8.3%)	0/10 (0%)	1.000
	negative	11 (91.6%)	10/10 (100%)	
CD23 negative		3/3 (100%)	1/1 (100%)	
cyclin D1 positive		12/12 (100%)	12/12 (100%)	
BCL2 positive		5/6 (83.3%)	6/6 (100%)	
BCL6 negative		7/7 (100%)	4/4 (100%)	
TdT negative		4/4 (100%)	2/2 (100%)	
Ki-67 proliferation index		Mean=58.09% Range: 40-90%	Mean=16.33% Range: 2-33%	0.005
p53 (n=6) (range=10 to 80%)		4 (>30% nuclear positivity nuclear positivity)		
		2 (<30%)		

### Treatment details

An anthracycline-based chemotherapy regimen, along with rituximab comprising cyclophosphamide, adriamycin, vincristine, and prednisolone (R-CHOP), was used as a front-line treatment. Table 4 highlights the treatment and follow-up status of the 12 patients with B-MCLs. The treatment approach was individualized based on patients’ affordability, clinical stage, commutability, and cooperation.

**Table 4.** Treatment and survival outcome of our cases (as of June 2023).

Patient No	Treatment	
1	BR *	Patient is alive and is under follow up
2	BR	Diagnosed with MCL in 2016 and relapsed in 2021 as BMCL. Died the same year
3	BR	Patient was diagnosed in 2021 and died in 2022
4	RCHOP **+BR	Patient was diagnosed as BMCL in the year 2021 and is under regular follow up
5	RCHOP+BR	Patient was diagnosed in the year 2017 and is under regular follow up
6	RCHOP+BR	Patient was diagnosed in the year 2014 as MCL. Relapsed in the year 2017 as BMCL, Died <1yr
7	RCHOP+BR	Diagnosed in the year 2017 and died in the year 2020
8	RCHOP	Patient diagnosed with BMCL in 2014 and is under regular follow up
9	RCHOP	Patient diagnosed in the year 2015 and deceased in the year 2020
10	RCHOP	Patient diagnosed in the year 2021 and died in the year 2023
11	Died prior to starting therapy	Patient was diagnosed as MCL an relapsed in the year 2016 for BMCL and died immediately before starting therapy. No data available on previous treatment history
12	Died prior to starting therapy	Relapsed case of MCL diagnosed in the year (2016) and diagnosed as BMCL in 2017. Died immediately before starting treatment. No data available on previous treatment history

* BR is Bendamustine and rituximab therapy.

** RCHOP stands for Rituximab+cyclophosphamide+dauxorubicin+Vincristine+prednisolone.

### Survival analysis

The log-rank for the data in our study was 0.887; thus, the two curves were not statistically significant. This might be due to the small sample size. The survival rates were also influenced by the COVID outbreak which occurred in 2020. The median overall survival for cases was 2 years, and for controls, it was 8 years ( Figure 4).

## Discussion

MCL usually occurs in the 7 ^th^ decade with a high male-to-female ratio and advanced clinical stage (stage III/IV). Lymphadenopathy is the most common presentation, but leukemic non-nodal (nnMCL) with bone marrow and splenic involvement has rarely been described. B-MCL, a morphological variant of MCL, is an aggressive subtype with a heterogeneous clinical course and a limited response to standard chemotherapy regimens. ^6^^,^^7^

Among the demographic and clinical characteristics, there were no major differences between B-MCL and classic MCL in our series. Extra nodal involvement was, however, more frequent in B-MCL (n=6, 50%), with GIT being the most common extra nodal site involved. CNS involvement in MCL is associated with elevated Mantle cell lymphoma International Prognostic (MIPI) score, elevated lactate dehydrogenase (LDH) levels, and high Ki-67 index of ≥30%, ^7^ suggesting transformation to a more aggressive subtype during relapse. The One case with CNS involvement in our series showed a high Ki-67 index of 70%, with markedly elevated serum LDH levels. On the contrary only 1/12 of the controls (8.33%) showed extra nodal involvement of GIT. This observation was statistically significant with a *p* value of 0.024. Extra nodal sites commonly affected in MCL include the gastrointestinal tract (lymphomatous polyposis), CNS, Waldeyer ring, spleen, and bone marrow. ^6^

**Figure 1.**
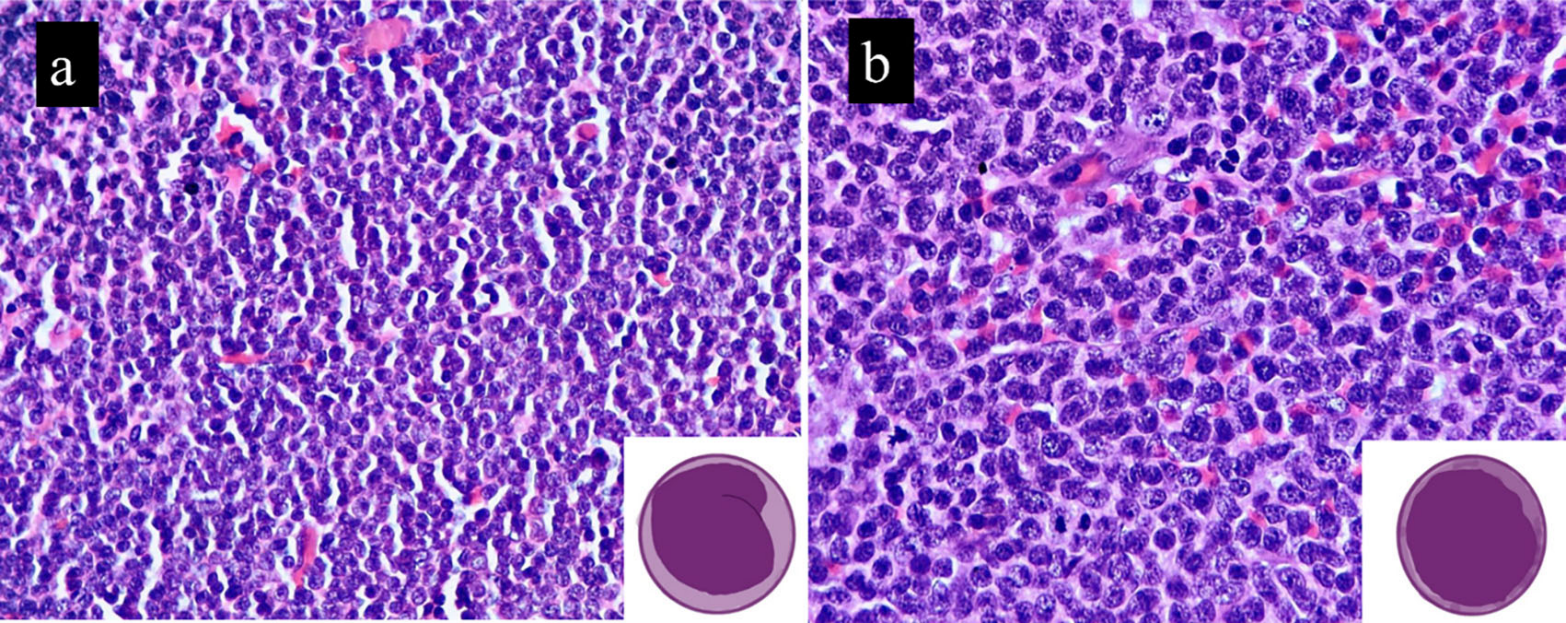
B-MCL cells are medium sized with immature chromatin and scant cytoplasm (b) when compared to classic variant (a). Inset depict the same. Image drawn using iPad pro.

During its course, classic MCL can progress into one of its variants, blastoid (B-MCL) or pleomorphic (P-MCL). ^1^^,^^2^^,^^6^^,^^9^ In this series, 4/12 cases (25%) of B-MCL arose via progression from classic MCL. Khanlari et al. opined that B-MCL and P-MCL are distinct aggressive variants that should be identified separately whenever possible. ^2^ They like Hoster et al. observed that Ki-67 index was significantly higher in B-MCLs (80%) vs P-MCLs (39%). Similarly, *NOTCH1* mutations are more common in B-MCL than in P-MCL. They also described another hybrid variant of MCL, with blastoid chromatin and a higher degree of pleomorphism. However, because it was more similar to B-MCL, they concluded that it was a variant of B-MCL. ^10^ Blastoid transformation is portended by leukocytosis, high LDH, high proliferation index, histomorphologic changes, diffuse growth pattern, increase in nuclear size, increase in mitosis, and pleomorphism, fulfilling the criteria of B-MCL/P-MCL ^1^^,^^6^ ( Figure 1). We had four cases which underwent transformation to B-MCL on relapse. One of our cases did show slight pleomorphism, but the Ki-67 was very high (>50%) and was thus called B-MCL.

On immunohistochemistry, MCLs express surface immunoglobulins (IgM, IgD), light chain restriction (lambda), pan B cell markers (CD19, CD20, CD79a), CD5, cyclin D1(>95%). Aberrant loss of CD5 and expression of CD10 and BCL6 are rare but are more common in aggressive variants. We had one case each of CD5 -ve and CD10+ve, while BCL6 was negative in all cases. In their extensive review of CD5 negative MCL, Soleimani et al. ^11^ observed improved survival in these patients independent of other favourable prognostic markers such as SOX11 loss and low Ki-67 and κ light chain restriction. Conversely, this advantage was lost in the presence of a blastoid morphology. Multitude genetic alterations seen in B-MCL (complex karyotype, somatic mutations involving vital genes – *TP53, NOTCH1*, *NOTCH2, CDKN2A*, and *MUC2* aberrations) were likely to overwhelm good prognostic factors.

Xu et al. ^12^ described the clinicopathological and prognostic significance of CD10 expression in 30 MCL cases. They opined that CD10 expression in the more aggressive subsets of MCL – high Ki-67>60%, blastoid/pleomorphic morphology, high MIPI, contributed to worse overall survival (OS) and was statistically significant with *p* value of <0.05. They also observed a diffuse growth pattern, blastoid/pleomorphic morphology, and BCL6 expression in these cases. ^12^ One of our B-MCL cases showed CD10 expression, with diffuse growth pattern and Ki-67 of 40%, but BCL6 was negative. The literature review showed variable BCL6 positivity in MCL ranging from 11 to 75%. ^13^^,^^14^

Cyclin D1 negative MCL cases lack cyclin D1 and *CCND1* rearrangements and may have *CCND2* rearrangements or genetic alterations leading to overexpression of cyclin D2 and cyclin D3 or rarely due to truncated cyclin D1 mRNA. ^15^ SOX11 (>90%) helps to identify CD5 and cyclin D1 negative cases. However, it is not specific for MCL and is also positive in lymphoblastic lymphoma, hairy cell leukemia, and Burkitt lymphoma. ^14^ In this study, we only included cases that were cyclin D1 positive, as SOX11 was not available in our setup. This is probably a limitation of this study.

MCL is generally associated with an aggressive, albeit heterogeneous, clinical course, inadequate response to chemotherapy, and a high recurrence rate with poor long-term prognosis. The blastoid and pleomorphic variants behave even more aggressively, with a median overall survival of 18 months. ^16^ The median OS for cases was 2 years and that for controls was 8 years in this study. Out of 12 cases, 7 cases were dead at the time of this study, with OS ranging from 1 month to 10 years. All four patients who progressed from classic MCL died within a year of B-MCL diagnosis. Mortalities were also observed in the control group (5/12), with four cases clustered around 2020 (peak COVID time in India).

MCL is considered an incurable disease; however, with newer therapeutic approaches, survival has increased to a few years. The prognostic markers for MCL as proposed by WHO for current clinical use incorporates: Age, performance status, CNS involvement at diagnosis, Stage (I, II vs III, IV), S. β2 microglobulin, S. LDH, morphology (Classic vs Blastoid). MCL international prognostic index (MIPI), Ki-67 index (<30% vs >30%), p53 by IHC, *TP53* deletions/mutations by sequencing analysis. ^6^ The MIPI is a tailored prognostication index for MCL, based on four independent prognostic factors: age, performance status, LDH, and leukocyte count. ^10^ We could not evaluate MIPI in this study, as none of the necessary factors were documented in the patient files.

**Figure 2.**
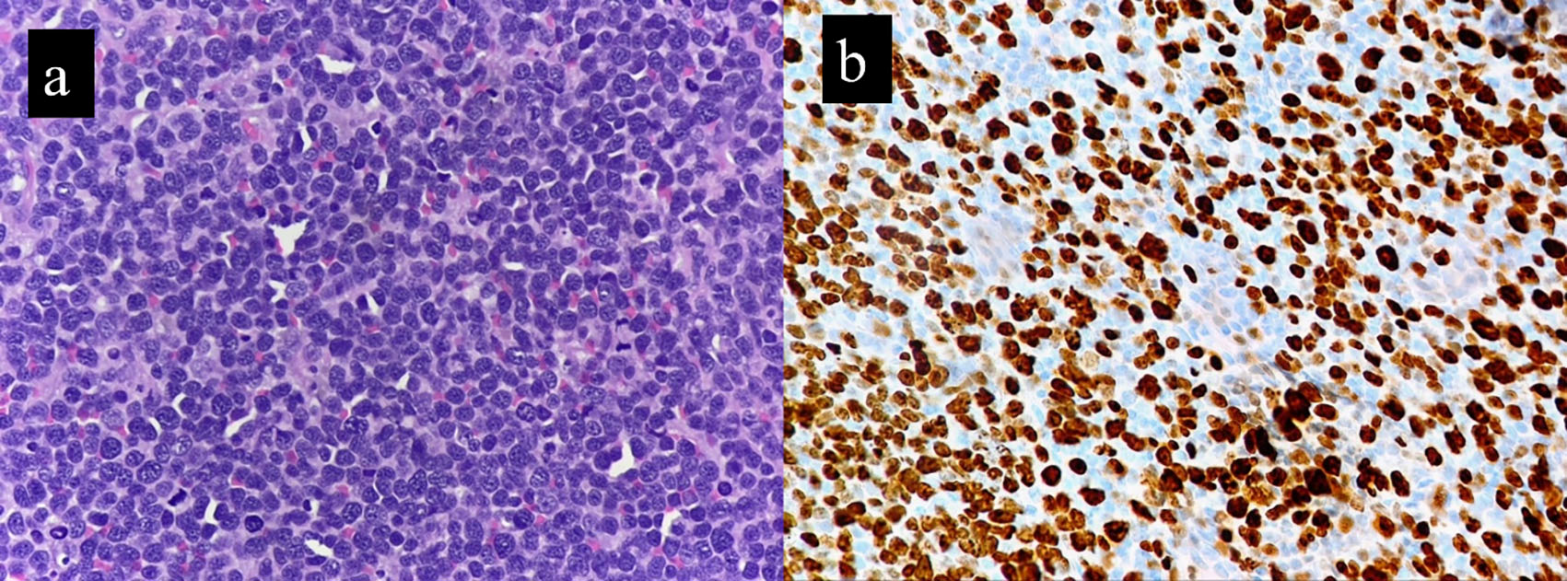
B-MCL exhibits high mitotic activity (a) which is reflected on Ki-67 IHC immunostaining (b).

The Ki-67 proliferation index has additional prognostic relevance in MCL. ^10^ A Ki-67 index >30% is associated with worse outcomes. ^17^ Most of our cases had very high Ki-67 index, in the range of 40-90% ( Figure 2). Several studies have reported p53 IHC expression in mantle cell lymphomas. The percentage cut off for p53 overexpression as determined by IHC varies across different studies, ranging from 10 to 30 to 50%. ^5^^,^^8^^,^^18^ In our study, two out of six cases showed low p53 expression. A cut-off of 30% was taken as per Nordic lymphoma study group. ^8^ A p53 IHC expression of more than 50% is associated with poor overall survival of less than 2yrs. A complex karyotype, *MYC* translocation or overexpression, and unmutated IGHV status are additional poor prognostic markers. ^5^^,^^18^

**Figure 3.**
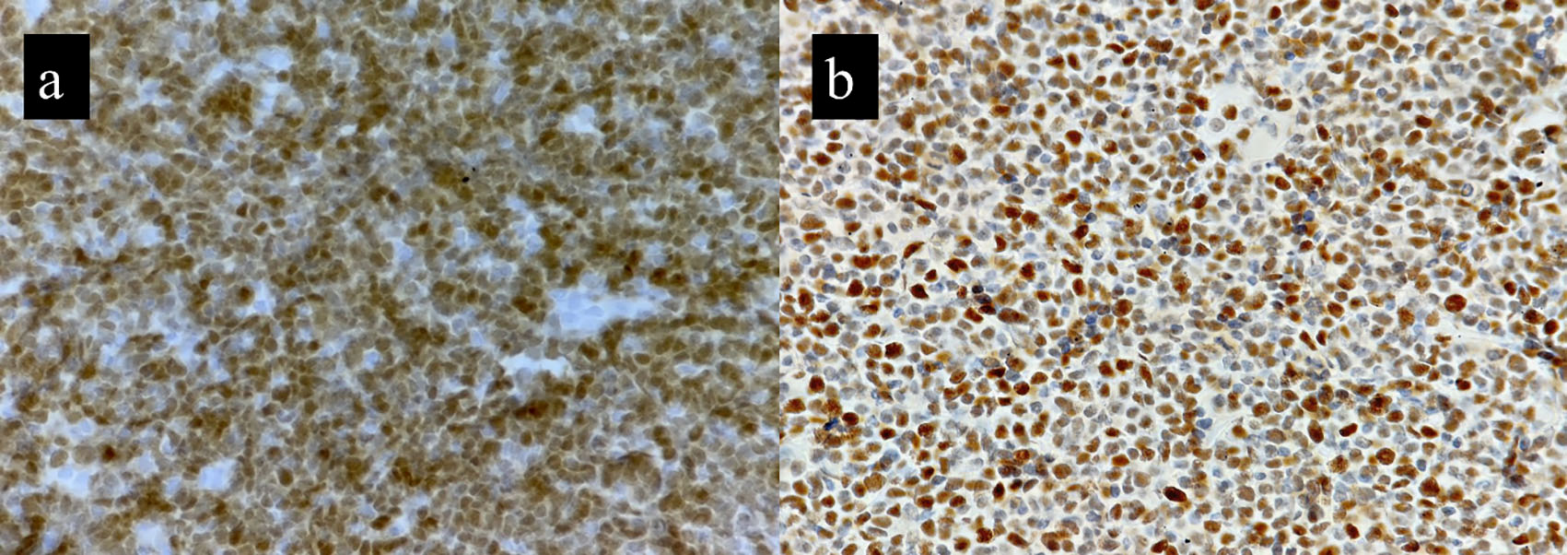
Cyclin D1 immunopositivity in BMCL (a) and p53 IHC showing >30% expression (b).

**Figure 4.**
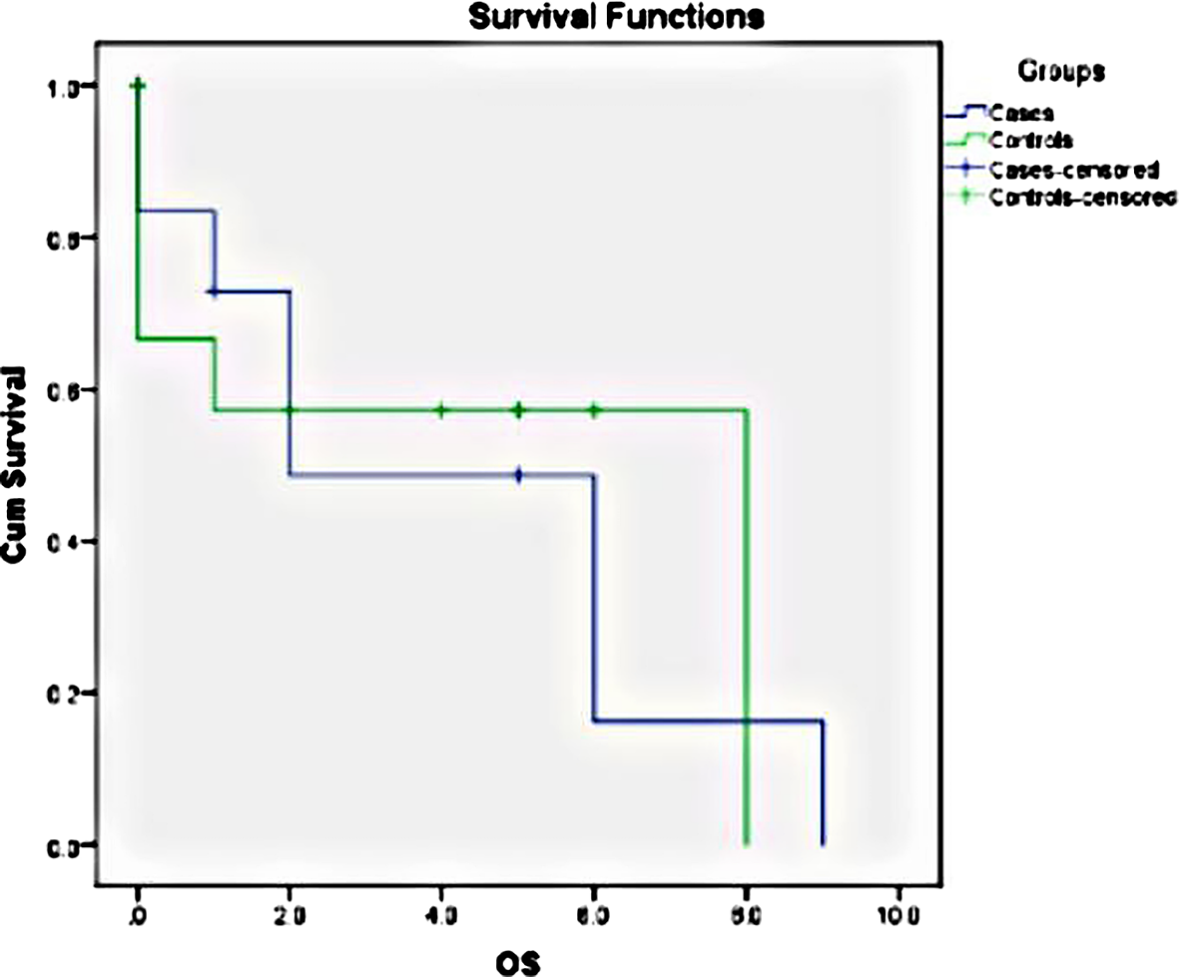
The Kaplan-Meier estimator was used to estimate the survival function, and it shows the probability of survival in time interval. The survival comparison between cases and controls by using Log-rank method showed no statistical significance in survival with Chi-Square value = 0.020 and p-value = 0.887.

These are some of the differential diagnoses that we need to consider when dealing with B-MCL; Diffuse large B-cell lymphoma (DLCL) can have cells that resemble blastoid cells of B-MCL. However, they can be ruled out by their negativity for CD5 and cyclin D1. Few cases of DLBCL can express cyclin D1(2%), but they are negative for SOX11. ^2^^,^^19^ Lymphoblastic lymphomas must be ruled out because of their close resemblance to the blastoid cells of B-MCL. Nuclear positivity for TdT helps to do the same. It is important to remember that plasma cell myeloma, blastic variant can be cyclin D1 positive (30-40%). However, plasma cells are CD5 and CD20 negative with CD138 positive. ^20^ Occasionally, a starry sky appearance might be observed in Burkitt lymphoma, which is positive for CD10 and BCL6 and negative for CD5 and cyclin D1. ^6^

There is no established treatment regimen for BMCL. Combined conventional doses of polychemotherapy followed by involved field radiotherapy are available for clinical use. Since MCL patients usually present at an advanced stage, systemic therapy is the standard treatment, with rare surgical intervention in cases presenting with massive splenomegaly or bowel obstruction. However, the response rate with anthracycline based CHOP regimens (cyclophosphamide, doxorubicin, vincristine, and prednisone) was much lower (13-50%) than that of other lymphomas. ^21^ Addition of rituximab (R-CHOP) has been linked to improved survival. Intensive immunochemotherapy with/without allogeneic stem cell transplantation in first remission followed by maintenance and/or consolidation therapies with anti-CD 20 antibodies and/or novel agents including bortezomib, ibrutinib, and lenalidomide have been proposed to improve progression-free and overall survival. ^5^^,^^22^

## Conclusion

In summary, our study demonstrates the poor prognosis of B-MCL patients with higher extra nodal involvement compared to the classic variant. The diagnosis of B-MCL requires meticulous morphological evaluation coupled with comprehensive immunohistochemistry panel. This amalgamation is required for accurately diagnosing B-MCL and to ruling out its morphological mimics. Immunohistochemical variations can be present leading to diagnostic confusion. Furthermore, due to aggressive nature of B-MCL, prompt and tailored therapeutic interventions become imperative. Rarity, aggressiveness, morphological, and immunohistochemical variations with a lack of standard treatment regimens make B-MCL a diagnostic and therapeutic challenge.

### Limitations

The study did not include cyclin D1 negative cases (non-availability of SOX11). The *p* value might not be representative because of the low number of cases. Hence, larger study groups are recommended for appropriate values.

#### Ethical considerations

The study has been approved by the Kasturba Medical College and Kasturba Hospital Institutional Ethics Committee (IEC) (Ref: IEC1:325/2023) with date of approval 18/10/2023.

## Consent

As the study is retrospective does not involve any intervention of subjects and uses lab based coded data collection; Consent waived by the ethics committee.

## Data Availability

Figshare: Clinical and pathological characteristics of Blastoid mantle cell lymphoma: a single institution experience. DOI: https://doi.org/10.6084/m9.figshare.25437115.v3. ^23^ The project contains the following underlying data:
• BMCL.xlsx. (Anonymised excel sheet of clinical, pathological, immunohistochemical and treatment aspects of cases and controls) Data are available under the terms of the Creative Commons Attribution 4.0 International license (CC-BY 4.0). Figshare: STROBE-checklist: Clinical and pathological characteristics of Blastoid mantle cell lymphoma: A single institution experience. DOI: https://doi.org/10.6084/m9.figshare.25700322.v5. ^24^ Data are available under the terms of the Creative Commons Attribution 4.0 International license (CC-BY 4.0).
