# Supplementary material for: Clinical and pathological characteristics of blastoid mantle cell lymphoma: a single institution experience
Source: F1000Res. 2024 Jul 26;13:525. Originally published 2024 May 22. [Version 2] doi: 10.12688/f1000research.149582.2 (PMC11297385; doi:10.12688/f1000research.149582.2)
Supplement: Peer review history

# Reviewer report: Reviewer response for version 2

**DOI:** 10.5256/f1000research.169467.r307401

**Published:** 2024-08-02

**Authors:** Vijay Shankar S

**Affiliations:** 1 Adichunchanagiri Institute of Medical Sciences, Adichunchanagiri University, Mandya, Karnataka, India

Queries addressed. No further comments.

Is the work clearly and accurately presented and does it cite the current literature?

Yes

If applicable, is the statistical analysis and its interpretation appropriate?

I cannot comment. A qualified statistician is required.

Are all the source data underlying the results available to ensure full reproducibility?

Yes

Is the study design appropriate and is the work technically sound?

Yes

Are the conclusions drawn adequately supported by the results?

Yes

Are sufficient details of methods and analysis provided to allow replication by others?

Yes

Reviewer Expertise:

Pathology

I confirm that I have read this submission and believe that I have an appropriate level of expertise to confirm that it is of an acceptable scientific standard.

# Reviewer report: Reviewer response for version 1

**DOI:** 10.5256/f1000research.164062.r287306

**Published:** 2024-06-19

**Authors:** Suma Mysore Narayana

**Affiliations:** 1 Department of Pathology, Kidwai Memorial Institute of Oncology (KMIO), Bangalore, Karnataka, India

A good study. I congratulate the authors for the conceptualization of this study

 I have few queries. 

- What was the total number of MCL in 10years. Random 12 cases are taken as control, was there any criteria to select them.
- was there Cyclin D1 negative mantle lymphoma picked up with SOX11.
- was curious to know  regarding the decision of regimens to treat MCL- was one superior over the other, BR vs R- CHOP

Is the work clearly and accurately presented and does it cite the current literature?

Yes

If applicable, is the statistical analysis and its interpretation appropriate?

Yes

Are all the source data underlying the results available to ensure full reproducibility?

Partly

Is the study design appropriate and is the work technically sound?

Yes

Are the conclusions drawn adequately supported by the results?

Yes

Are sufficient details of methods and analysis provided to allow replication by others?

Yes

Reviewer Expertise:

oncopathology with special interest in female genital tract tumors, Breast tumors, MGT tumors, endocrine tumors

I confirm that I have read this submission and believe that I have an appropriate level of expertise to confirm that it is of an acceptable scientific standard.

# Response

**Published:** 2024-07-17

**Authors:** SWATHI PRABHU

**Affiliations:** Pathology, Manipal Academy of Higher Education, Manipal, Karnataka, India

Thank you for sparing your valuable time to review our article. We appreciate time and effort in providing feedback on our manuscript and are grateful for the insightful comments.

 Reviewer: What was the total number of MCL in 10years. Random 12 cases are taken as control, was there any criteria to select them

 Author response: There were 21 cases totally, random 12 cases were selected as controls. 

 Reviewer: Was there Cyclin D1 negative mantle lymphoma picked up with SOX11?

 Author response: No. SOX11 is not available in our facility.  We agree that this is a potential limitation of the study. We have added this as a limitation in our manuscript.

 Reviewer: was curious to know  regarding the decision of regimens to treat MCL- was one superior over the other, BR vs R- CHOP

 Response: There is not much difference in OS of patients treated with BR therapy and R-CHOP. The study involves a small cohort of patients, and the prognosis of mantle cell lymphoma (MCL) itself is poor. Hence, determining which regimen is better is difficult in this study. Clinical trials have shown that BR can be as effective as R-CHOP in terms of response rates

# Reviewer report: Reviewer response for version 1

**DOI:** 10.5256/f1000research.164062.r281826

**Published:** 2024-06-08

**Authors:** Vijay Shankar S

**Affiliations:** 1 Adichunchanagiri Institute of Medical Sciences, Adichunchanagiri University, Mandya, Karnataka, India

Dear Authors

 The manuscript is well-written and provides valuable insights into B-MCL. 

 However some of the below points to be addressed to strengthen the study

 a. Provide more details on how missing data and potential confounders were handled ( Eg Table 3)

 b. Provide clear and descriptive legends for the figure 4

 c. Discussion:  para3 , Line 3 sentence not grammatically correct.

Is the work clearly and accurately presented and does it cite the current literature?

Yes

If applicable, is the statistical analysis and its interpretation appropriate?

I cannot comment. A qualified statistician is required.

Are all the source data underlying the results available to ensure full reproducibility?

Yes

Is the study design appropriate and is the work technically sound?

Yes

Are the conclusions drawn adequately supported by the results?

Yes

Are sufficient details of methods and analysis provided to allow replication by others?

Yes

Reviewer Expertise:

Pathology

I confirm that I have read this submission and believe that I have an appropriate level of expertise to confirm that it is of an acceptable scientific standard, however I have significant reservations, as outlined above.

# Response

**Published:** 2024-07-21

**Authors:** SWATHI PRABHU

**Affiliations:** Pathology, Manipal Academy of Higher Education, Manipal, Karnataka, India

We authors, thank you for sparing your time in reviewing our article. We appreciate the time and efforts dedicated by you for providing feedback on our manuscript and are grateful for the insightful comments. 

**Reviewer:** Provide more details on how missing data and potential confounders were handled ( Eg Table 3)

**Author response:** The Confounding factors with respect to IHC: As we do not have a set panel of IHC markers done for all cases [resource limited facility, patients from low socio-economic strata], usually IHC markers are requested as per individual case and differs amongst faculty; cases which were cyclin D1 & CD5 + were included in the study. We have accepted this limitation in the manuscript also. The lone case which was CD5 -ve was Cyclin D1 + (described in aggressive variants of MCL). Ki67 was done in all cases. The other markers which were done at the time of initial diagnosis are documented. P53 was the additional marker we did only on the cases (financial constraints) upon start of this study. Similarly in Table 1, only details mentioned in the EMR are documented. We had no sources to get data that were not documented. Drawbacks of retrospective nature of the study.

**Reviewer :** Provide clear and descriptive legends for the figure 4.

**Author response:** Thank you for this suggestion. We have updated the same in our new version of the manuscript.

**Reviewer :** Discussion:  para3 , Line 3 sentence not grammatically correct. I didn’t get what this is

**Author response: **Thank you for pointing out this mistake. The sentence has been revised in our new version of the manuscript.
